# Slowly Repaired Bulky DNA Damages Modulate Cellular Redox Environment Leading to Premature Senescence

**DOI:** 10.1155/2020/5367102

**Published:** 2020-02-10

**Authors:** Yujie Zhang, Peiyan Guo, Wanchen Xiang, Qingxi Liu, Xinyi Liu, Ning Ma, Sa Zhou, Hongpeng He, Meinhard Wlaschek, Karin Scharffetter-Kochanek, Tong-Cun Zhang, Wenjian Ma

**Affiliations:** ^1^College of Biotechnology, Tianjin University of Science and Technology, Tianjin 300457, China; ^2^Qilu Institute of Technology, Shandong 250200, China; ^3^Department of Dermatology and Allergic Diseases, Ulm University, Albert-Einstein-Allee 23, 89081 Ulm, Germany; ^4^Institute of Biology and Medicine, Wuhan University of Science and Technology, Wuhan 430081, China

## Abstract

Treatments on neoplastic diseases and cancer using genotoxic drugs often cause long-term health problems related to premature aging. The underlying mechanism is poorly understood. Based on the study of a long-lasting senescence-like growth arrest (10-12 weeks) of human dermal fibroblasts induced by psoralen plus UVA (PUVA) treatment, we here revealed that slowly repaired bulky DNA damages can serve as a “molecular scar” leading to reduced cell proliferation through persistent endogenous production of reactive oxygen species (ROS) that caused accelerated telomere erosion. The elevated levels of ROS were the results of mitochondrial dysfunction and the activation of NADPH oxidase (NOX). A combined inhibition of DNA-PK and PARP1 could suppress the level of ROS. Together with a reduced expression level of BRCA1 as well as the upregulation of PP2A and 53BP1, these data suggest that the NHEJ repair of DNA double-strand breaks may be the initial trigger of metabolic changes leading to ROS production. Further study showed that stimulation of the pentose phosphate pathway played an important role for NOX activation, and ROS could be efficiently suppressed by modulating the NADP/NADPH ratio. Interestingly, feeding cells with ribose-5-phosphate, a precursor for nucleotide biosynthesis that produced through the PPP, could evidently suppress the ROS level and prevent the cell enlargement related to mitochondrial biogenesis. Taken together, these results revealed an important signaling pathway between DNA damage repair and the cell metabolism, which contributed to the premature aging effects of PUVA, and may be generally applicable for a large category of chemotherapeutic reagents including many cancer drugs.

## 1. Introduction

DNA damage is known that can promote aging and age-related diseases. Deficiencies in DNA repair pathways like nucleotide excision repair (NER) and double-strand break repair (DSBR) have been well-established that can cause accelerated aging, and they are underlying some severe human genetic disorders such as Werner syndrome, xeroderma pigmentosum, and Cockayne syndrome [1, 2]. Premature aging can also be triggered by certain DNA damage reagents including drugs for chemotherapy [3, 4]. After decades of using genotoxic drugs in chemotherapy against cancer and other neoplastic diseases, a variety of side effects was observed that resemble accelerated aging, such as decline of cognitive functions, osteoporosis, chronic fatigue, and cardiovascular complications [5, 6].

It is easy to understand that genetically impaired DNA repair, due to their impacts on the whole organism including stem cells, can lead to persistent accumulation of DNA mutations and even deplete the pool of hematopoietic stem cells with age, therefore leading to premature aging [7–9]. However, the mechanism on how DNA damages induced by chemotherapeutic drugs, which impact part of the cells/tissue and are either repairable in normal cells or can be replaced by newly differentiated cells from stem cells, cause or contribute to progeroid functional decline is still poorly understood [2, 5].

Agents that induce DNA interstrand cross-links (ICLs) are one of the earliest and still the most widely used forms of chemotherapeutic drug [10]. ICL is a deadly type of DNA damage since it prevents transcription and replication by inhibiting DNA strand separation. Among the most potent interstrand cross-linking agents are psoralens, which form ICLs upon UVA photoactivation (PUVA) [5, 11]. PUVA is widely used for the treatment of various skin disorders, such as psoriasis and vitiligo [12, 13]. With antibodies that can specifically recognize psoralen photo adducts, earlier studies had demonstrated DNA as the major target of PUVA [14]. While psoralens can also form thymine monoadducts with DNA that contributed to the therapeutic effect, the formation of ICL plays an important role on the induced cytotoxicity [11, 15]. Although PUVA is effective and being used for many decades, it is associated with cumulative long-term risks, including premature skin aging and the increased occurrence of squamous cell carcinoma [11, 16].

We have previously shown that a single nontoxic exposure of human dermal fibroblasts to 8-methoxy psoralen plus UVA resulted in a growth arrest with morphology changes and gene expression pattern reminiscence of cellular senescence [17]. The growth arrest was extremely long-lasting which was more than two months; surprisingly, it was reversible and the cells restarted proliferation and seems identical to its presenescent state [18]. Here, using this PUVA-induced senescence-like growth arrest as a model, we studied the crosstalk between the DNA repair and cell metabolism to address the question on how a one-time drug treatment can cause a “molecular scar” leading to long-term unfavorable outcomes such as premature skin aging.

## 2. Materials and Methods

### 2.1. Cell Culture

Primary fibroblasts were established from the foreskin of healthy human donors aged 3 to 6 years. Cells were maintained in Dulbecco's modified Eagle's medium (DMEM) supplemented with 10% fetal calf serum (FCS). For determination of growth rates and cumulative population doublings (CPDs), cells were counted at each transfer with a Fuchs-Rosenthal haemocytometer.

### 2.2. PUVA Treatment

Fibroblasts were preincubated with 50 ng/ml of 8-methoxypsoralen (8-MOP) and cultured in DMEM for 16-18 hours, then irradiated in PBS at a dose of 90 kJ/m^2^ using a high-intensity UVA source (UVASUN 3000 equipped with the UVASUN safety filters to help transmit the desired wavelength, Mutzhas, Munich, Germany) emitting wavelengths in the 340-450 nm range. Fluencies were determined with a spectrum-adapted UVA-ultraviolet meter (Beckman UV 5270).

### 2.3. Telomere Length Analysis

Telomere length was analyzed by digest genomic DNA with restriction enzymes Hinfl/Rsal followed by Southern blot analysis using a 51-mer biotinylated telomeric probe ((TTAGGG)_8_-TTA). The mean TRF (terminal restriction fragment) length was estimated by comparing the size of the distributed fragment position to the molecular weight markers [19]; data showed are the representative images from more than 3 repeats.

### 2.4. Determination of Single-Strand DNA Breaks in Telomere

Genomic DNA isolated from PUVA-treated fibroblasts at different time points as well as mock-treated and regrown fibroblasts were digested first with RsaI/HinfI (1 U/*μ*l); part of the samples was treated further with alkaline buffer (0.4 M NaOH, 4 mM EDTA) for 10 min to denature DNA, then subjected to electrophoresis and Southern blot. Nondenatured RsaI/HinfI-digested DNA samples were used as control.

### 2.5. ROS Measurement

Levels of intracellular ROS were assessed by loading cells with 10 *μ*g/ml of a fluorescent ROS indicator 2′-7′ dichlorodihydrofluorescein diacetate (H_2_DCF-DA) (Sigma-Aldrich) for 20 min and followed by detection using a laser scanning confocal microscope. The excitation wavelength is 488 nm, and emission wavelength is 521 nm. The resulting image is an artificial color made by the computer program based on intensity of the emitted photons. All experiments were repeated at least 3 times.

### 2.6. Measurement of the Mitochondrial Mass and Membrane Potential

Mitochondrial mass was determined by staining living cells with 25 nM of the fluorescent dye MitoTracker Red CMXRos (MoBiTec, Goettingen) in DMEM for 30 min at 37°C, then photograph using a 568 nm line of fluorescence excitation with a laser scanning confocal microscope. To measure the mitochondrial membrane potential (*ΔΨ*_m_), PUVA-treated cells at different time points were washed and resuspended in PBS with 5 mg/ml of JC-9 (MoBiTec, Goettingen), a polarization-sensitive dye which exists in two interchangeable forms, monomer (green fluorescence, 530 nm) and dimer (red fluorescence, 590 nm) [20, 21]. After 20 min incubation at 37°C, cells were immediately analyzed by flow cytometry. Data were collected by analyzing an average population of 20,000 cells.

### 2.7. Enzyme Activity Analysis

NADPH oxidase activity was measured as previously described [22]. Briefly, control and PUVA-treated fibroblasts were scraped and lysed in phosphate buffer (20 mM, pH 7.0) containing 1 mM EGTA, 10 mM aprotinin, 0.5 mM leupeptin, 0.7 *μ*g/ml pepstatin, 0.5 mM phenylmethylsulfonyl fluoride, and 1x protease inhibitor mixture (Sigma). Total cell suspension (250 *μ*l) was mixed with 250 *μ*l HBSS containing 500 *μ*M lucigenin and kept at 37°C for 10 min. NADPH oxidase activity assay was initiated by adding 10 *μ*l of NADPH (100 *μ*M) as substrate and measured the absorbance at 595 nm on a plate reader spectrophotometer.

Glucose-6-phosphate dehydrogenase (G6PDH) activity was measured using a commercial kit by the colorimetric assay (Sigma-Aldrich) based on its catalyzing the conversion of glucose-6-phosphate to 6-phosphogluconate with reduction of NADP to NADPH. Briefly, cell extracts of PUVA-treated fibroblasts at different time points were added into G6PDH substrate mix containing glucose 6-phosphate and NADP+ in Tris-HCl buffer (pH 7.6) and incubate at 37°C for 5 minutes. G6PDH activity was determined based on the production of NADPH that reduces the blue dye dichlorophenolindophenol to its colorless form in the presence of phenazine methosulfate, and the absorbance at 450 nm was measured expressing as arbitrary unit/million cells.

### 2.8. Quantitative Real-Time RT-PCR (qRT-PCR)

Total RNA was isolated from PUVA-treated and control fibroblasts using Trizol reagent (Invitrogen); reverse-transcribed complementary DNA were synthesized with random primers. The primers used for qRT-PCR to detect NOX2, NOX4, ATM, BRCA1, and mtDNA were listed in supplemental [Supplementary-material supplementary-material-1]. qRT-PCR was performed using the Fast SYBR Green Master Mix (Applied Biosystems) with Biosystems StepOne^TM^ Real-Time PCR machine (Applied Biosystems, CA).

### 2.9. Western Blotting

Control and PUVA-treated fibroblasts were lysed using RIPA buffer, and total proteins were separated by SDS-PAGE followed by Western blot. The following primary antibodies were used: rabbit anti-PP2A (Bioss), BRCA1 (Proteintech), NOX2, NOX4, ATM, 53BP1 (Abcam), and mouse anti-*β*-actin (Santa Cruz Biotechnology). The secondary antibodies used were IRDyeTM-800-conjugated anti-mouse or anti-rabbit IgG (LI-COR Biosciences). Immunoreactivity was detected using an Odyssey Infrared Imaging System (Gene Company Limited). All immunoblots were repeated at least 2-3 times.

## 3. Results

### 3.1. A Single PUVA Treatment of Human Dermal Fibroblasts Led to Long-Lasting Growth Arrest and Sustained Generation of Intracellular Reactive Oxygen Species

**Figure 1:**
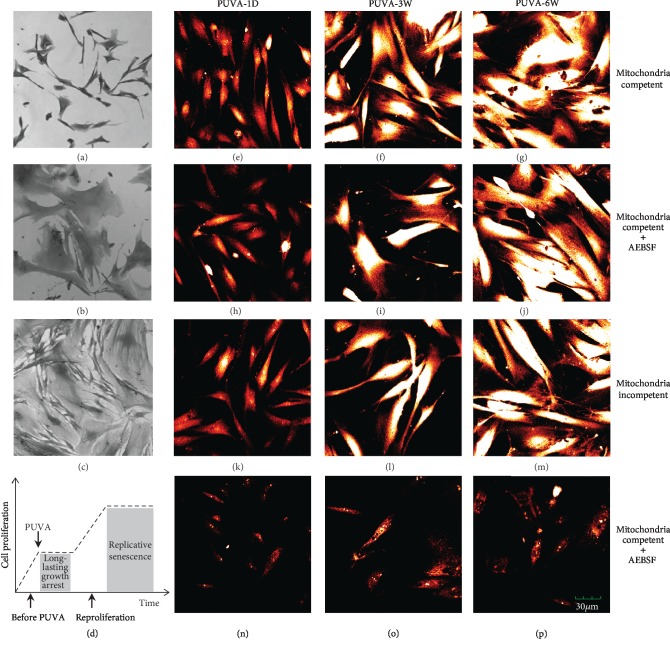
PUVA treatment induced long-lasting growth arrest and persistent ROS production from mitochondria and NADPH oxidase. (a) Mock-treated control fibroblast; (b) growth arrested fibroblasts at week 6 after PUVA; (c) fibroblasts start to reproliferate after 10-12 weeks post-PUVA treatment; (d) research model: ROS signaling during the growth arrest stage, and PUVA's impacts on replicative senescence were addressed during the reproliferation stage. (e–p) ROS level determination by DCF at different time points (1 day, 3 weeks, and 6 weeks) post-PUVA treatment. Fluorescence intensity was recorded with a laser scanning confocal microscope (×100 magnification) with artificial red color. NOX inhibitor AEBSF (100 *μ*M, added to PUVA-treated fibroblasts for 2 hours before DCF staining). All fluorescence images were representatives of at least 3 independent measurements.

Upon a single PUVA treatment with clinical relevant dose (50 ng/ml of 8-MOP plus 90 kJ/m^2^ UVA), human dermal fibroblasts were growth arrested with functional changes similar to cellular senescence such as the increased expression of metalloproteinase-1 (MMP-1) and SA-*β*-galactosidase [17, 18]. Cells became much enlarged as compared to mock-treated control fibroblasts (Figures 1(a) and 1(b)). Although the growth arrest was long-lasting (>2 months), cells have the full capacity of recovering from this growth arrest stage and generally started to proliferate again at ~10-12 weeks (Figure 1(c)). Using this uncommonly long-lasting growth arrest as a model, here, we studied the cellular signaling upon genotoxic drug-induced cellular senescence (SIPS) and its potential impact to aging. Figure 1(f) is a plot of different cell stages following PUVA treatment where specific studies were pursued.

### 3.2. ROS Production Was due to the Activity of Both Mitochondria and NADPH Oxidase

An enhanced and persistent generation of ROS was observed during the whole growth arrest period as determined by the ROS indicator dichlorodihydrofluorescein diacetate (DCF), a peroxide-sensitive fluorophore [23]. Comparing to mock-treated fibroblasts, DCF fluorescence in PUVA-treated fibroblasts became evident at 24 hours posttreatment and time-dependently increased to >20-fold at 6 weeks (Figures 1(e)–1(g)). As the major source of ROS in the cell, we first asked whether the high ROS level following PUVA treatment was due to the malfunction of mitochondria considering that the possible formation of psoralen-DNA adducts with mtDNA may impact the integrity of mitochondrial oxidative phosphorylation. Therefore, mtDNA-depleted *ρ*0 fibroblasts were generated with ethidium bromide and were confirmed by measuring the expression of mitochondrial transcription factor mtTFA that had been shown to vary concomitantly with the mtDNA level (supplemental [Supplementary-material supplementary-material-1]) [24, 25]. Interestingly, PUVA-treated *ρ*0 fibroblasts still showed high ROS levels (Figures 1(k)–1(m)). Therefore, ROS is not only generated in mitochondria or due to mtDNA damage.

To further investigate the sources of ROS, we screened other cellular components that can generate ROS and identified that NADPH oxidase (NOX) played a critical role. Although the ROS level could not be suppressed by the inhibition of NOX with its inhibitors 4-(2-aminoethyl)-benzenesulfonyl fluoride (AEBSF) or diphenyleneiodonium (DPI) in mitochondrial competent fibroblasts (Figures 1(h)–1(j)), it was completely blocked when the NOX inhibitors were applied on *ρ*0 fibroblasts (Figures 1(n)–1(p)) (AEBSF and DPI had similar impacts, shown are the results of AEBSF as representative). These results demonstrated that PUVA-induced ROS production was from both mitochondria and NADPH oxidase.

### 3.3. ROS Generation Correlated with the Repair of Bulky DNA Damages and Could Be Partially Suppressed by the Combined Inhibition of DNA-PK and PARP1

The persistent production of ROS suggests that it is likely a secondary effect in response to PUVA-induced DNA damages. This was further studied by using reagents that induce different types of DNA damages. As shown in supplemental [Supplementary-material supplementary-material-1], ROS could also be induced by other cancer drugs such as cisplatin and doxorubicin. Similar to PUVA, cisplatin can form interstrand crosslinks, but intrastrand crosslinks are its major adducts whereas PUVA induces monoadducts [26]. Doxorubicin interacts with DNA by intercalation as well as inhibiting the progression of topoisomerase II [27]. Both drugs induce bulky DNA damages that normally require the coordination of various repair pathways for repair, such as nucleotide excision repair (NER), translesion DNA synthesis (TLS), and homologues recombination [10, 27]. In contrary, the level of ROS was much lower when fibroblast was treated with methyl methanesulfonate (MMS), a regent inducing DNA damages that are normally repaired by base excision repair (BER) [28]. These results suggest that ROS production could be significantly triggered only by DNA damaging reagents that generate ICL and similar bulky DNA damages.

**Figure 2:**
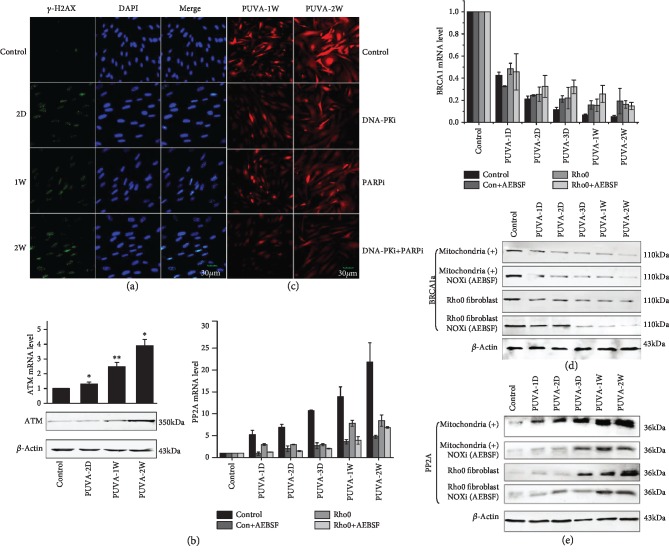
The correlation between PUVA-induced ROS and DNA damage repair. (a) Persistent formation of DSB as determined by H2AX foci from day 2 to week 2 post-PUVA; (b) ATM was upregulated after PUVA treatment and showed a positive correlation with ROS elevation. (c) PUVA-induced ROS could be partially suppressed by combined treatment with DNA-PK and PARP inhibitors (NU7026 and olaparib, respectively); (d) the expression of BRCA1 was negatively related to the level of PUVA-induced ROS, and it was not affected by NOX inhibition; (e) PUVA led to upregulation of PP2A that can be suppressed by NOX inhibition. All fluorescence images or Western blots were representatives of at least 3 independent measurements/repeats.

We then studied whether ROS production was related to specific DNA repair pathways, particularly those involved in ICL and DSB repair. As shown in Figure 2(a), PUVA induced the formation of *γ*-H2AX foci which kept a steady level or slightly increased from day 2 to week 2, suggesting there was DSB formation continually. This was further confirmed by the elevated expression of ATM (Figure 2(b)), a protein kinase that is primarily activated by DSBs [29]. DSB repair depends on two main pathways: homologous recombination (HR) and nonhomologous end joining (NHEJ). Although ICL requires homologous recombination for repair [10], the transcription and expression level of a key gene involved in HR processing, BRCA1, was reducing with time following PUVA treatment (Figure 2(d)), which is negatively correlated with ROS production. On the other hand, inhibition of NHEJ, the most commonly used pathway which signals DSBs through the Ku70/Ku80 heterodimer and DNA-dependent protein kinase (DNA-PK), showed some effect on the ROS level. Using a DNA-PK inhibitor NU7026, our data indicated that ROS could be partially inhibited as shown in Figure 2(c). There are also redundant pathways called Alt-NHEJ and microhomology-mediated end joining (MMEJ), which serves as a back-up to NHEJ though less efficient in repair [30, 31]. As an important player involved in Alt-NHEJ, the impact of PARP1 on ROS production was determined by its potent inhibitor olaparib. Incubation of PARP1 also showed minor inhibition on PUVA-induced ROS which was at a similar level as that of DNA-PK inhibition (Figure 2(c)). However, combined use of both DNA-PK and PARP inhibitors, the ROS level could be evidently suppressed. These results suggest that ROS production has some relation with the process of NHEJ.

The involvement of NHEJ instead of HR for ROS production was also supported by the increased expression of protein phosphatase 2A (PP2A) and 53BP1. PP2A is known that enhances Ku and DNA-PK activities to facilitate DSB repair as well as regulating ATM and DNA damage response network [32, 33]. As shown in Figure 2(e), PP2A mRNA and protein level were both elevated in PUVA-treated fibroblasts showing a positive correlation with ROS. Its expression was suppressed when NADPH oxidase was inhibited, but not in *ρ*0 cells. Consistent with previous findings [34], the level of pp2A was negatively correlated with BRCA1 (Figure 2(d)) in PUVA-treated fibroblasts. Together with the upregulation of 53BP1 (supplemental [Supplementary-material supplementary-material-1]) which is a key player controlling the repair choice between NHEJ and HR by suppressing the activity of BRCA1 [35], these data suggest the repair of DSB could change the cell's redox environment and being regulated vice versa. Extensive further studies are required to understand the underlying signaling cascade and regulation mechanisms.

### 3.4. Stimulation of Pentose Phosphate Pathway Is Responsible for NOX Activation and PUVA-Induced ROS Could Be Suppressed by NADP^+^ and Ribose-5-Phosphate

**Figure 3:**
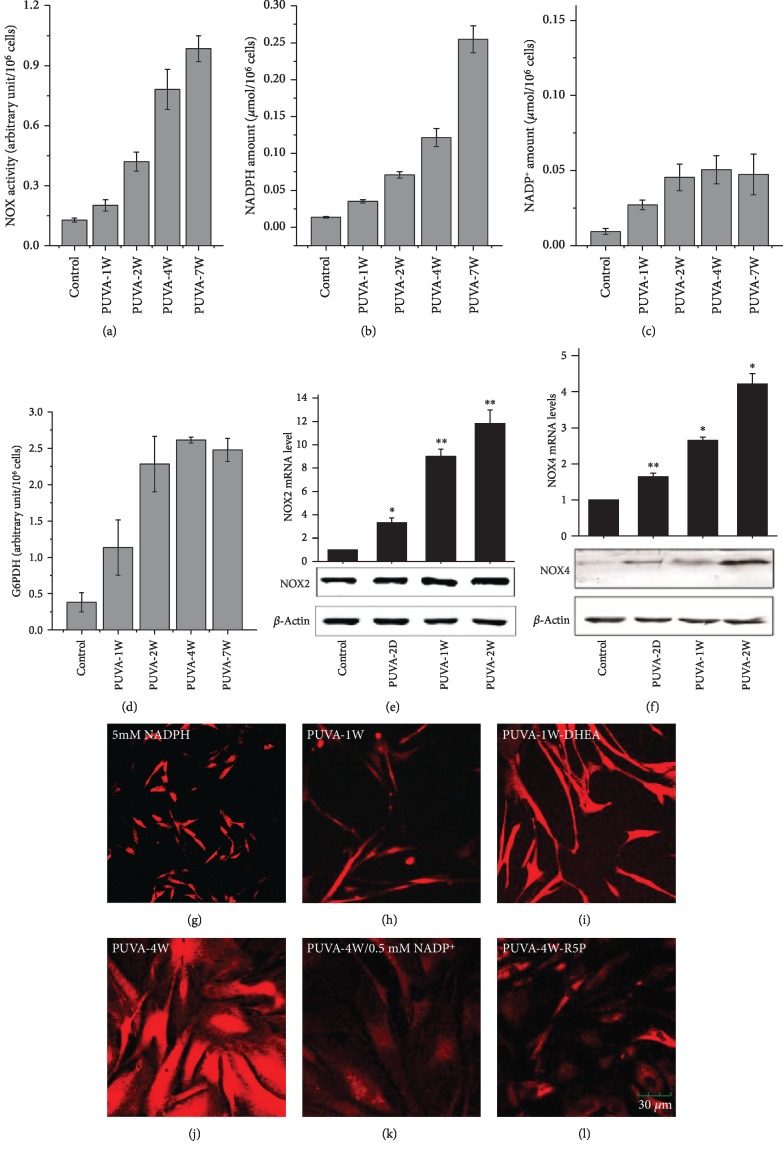
Increased NOX activity, NADPH level, and PPP stimulation following PUVA treatment. (a–d) At different time points after PUVA treatment, the enzyme activity of NOX (a) and G6PDH (d) and relative levels of NADPH (b) and NADP^+^ (c) were determined in vitro using cell lysates. The activity of NOX and G6PDH was represented as relative light emission units/min/10^6^ cells. All measurements are shown as the means ± SD from three independent experiments. (e, f) The mRNA and protein expression levels of NOX2 and NOX4 following PUVA treatment. (g–l) ROS determination by DCF; (g) control fibroblasts treated with 5 mM NADPH for 2 hours; (h, i) PUVA-treated fibroblasts at 1 week posttreatment without (h) or with (i) preincubation of G6PDH inhibitor DHEA (100 *μ*M) for 2 hours followed by DCF staining; (j) PUVA-treated fibroblasts (4 weeks); (k, l) PUVA-treated fibroblasts (week 4) with preincubation of NADP^+^ (0.5 mM) (k) or ribose-5-phosphat (R5P, 5 mM) for 2 hours followed by DCF staining.

NADPH oxidase is critical for redox signaling and maintaining the balance between cellular levels of oxidizers and reducers such as the endogenous redox pairs NADH/NAD^+^ and NADPH/NADP^+^ [36]. To further understand the mechanism underlying the PUVA-induced ROS, we determined the time course of NADPH oxidase activation as well as the related metabolic changes. As shown in Figure 3(a), the activity of NADPH oxidase was elevated during growth arrest which linearly correlated with the level of ROS. Consistently, the expression of NOX2 and NOX4, two major NADPH oxidase isoforms that generates H_2_O_2_ [37], was both elevated on their mRNA level. The NOX4 protein level increased by 4.3 times at 2 weeks post-PUVA treatment, but the NOX2 protein level was only slightly increased which is about 1.6 times (Figures 3(e) and 3(f)).

**Figure 4:**
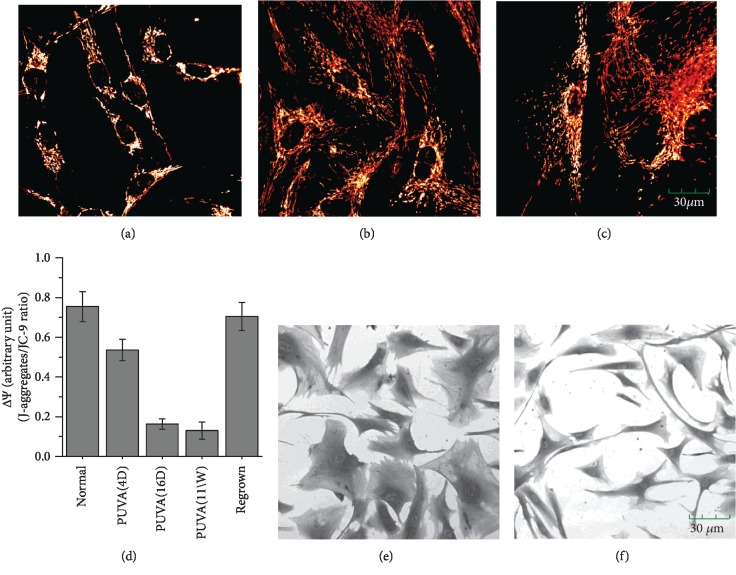
PUVA-induced mitochondrial mass increase and the suppression by ribose-5-phosphate. (a–c) The mitochondrial mass in control (a) and PUVA-treated (at week 3 and week 6) fibroblasts (b, c) determined by MitoTracker Red; (d) determination of mitochondrial membrane potential (*ΔΨ*) by a polarization-sensitive fluorescent dye JC-9 based on the ratio between two *ΔΨ*-driven interchangeable forms, monomer (J-monomer, green) and dimer (J-aggregate, red); (e, f) the cell morphology after PUVA (at week 3) without (e) and with ribose-5-phosphate (5 mM, f).

To understand how NOX was stimulated, we further investigated the participation of the pentose phosphate pathway (PPP), which generates NADPH and the 5-carbon pentose from glucose. We first measured the cellular level of NADPH and NADP+. As shown in Figures 3(b)–3(d), although both are elevated after PUVA treatment, the increase rate of NADPH was much higher based on the NADPH/NADP+ ratio, which showed a steady increase from ~1.3 in control fibroblasts and PUVA-1W to ~5.4 at PUVA-7W (Figures 3(b) and 4(c)). The increased NADPH/NADP+ ratio is in line with the elevation of the ROS level, suggesting it might be a direct reason to activate NOX. This is supported by the fact that treating normal fibroblasts with high concentration of NADPH did generate ROS (Figure 3(g)), which could be prevented by preincubation with NOX inhibitors (Supplemental [Supplementary-material supplementary-material-1]). The involvement of the PPP was confirmed by the increased activity of glucose-6-phosphate dehydrogenase (G6PDH) (Figure 3(d)), a rate-limiting enzyme that reduces NADP^+^ to NADPH while oxidizing glucose-6-phosphate [38]. Although the time course of the increased activity of G6PDH was also in line with the level of ROS, its inhibition by a specific inhibitor, DHEA, could not attenuate the ROS level (Figures 3(h) and 3(i)). This might be due to the redundant role of mitochondria in generating ROS when the PPP was blocked. Interestingly, feeding PUVA-treated cells with NADP^+^ or ribose-5-phosphate (R5P), a downstream product of the PPP and a precursor for nucleotide biosynthesis, could evidently suppress the ROS levels (Figures 3(k) and 3(l)). These data indicated that stimulation of the PPP for biosynthesis of certain metabolites was underlying the ROS production after PUVA treatment.

### 3.5. Increased Mitochondrial Biogenesis and Its Suppression by Feeding Cells with Ribose-5-Phosphate

Considering that NOX and PPP activation might also be related to defective mitochondria, the mitochondrial integrity was assessed after PUVA treatment by measuring its membrane potential (*ΔΨ*_m_) using a polarization-sensitive dye JC-9 [21]. In cells with intact mitochondrial membranes, JC-9 is driven into mitochondria by high *ΔΨ*_m_ to form dimmers (red fluorescence). As shown in Supplemental [Supplementary-material supplementary-material-1], FACS analysis revealed that the monomer form (green fluorescence) was evidently increased in PUVA-treated fibroblasts by 2 weeks (PUVA-16d) and further increased by 30-fold at 11 weeks (PUVA-11W) post-PUVA treatment comparing to that of control cells, indicating a general increase of mitochondrial mass. Although the dimer form per cell was also increased after PUVA, the dimer/monomer ratio, which represents the average *ΔΨ*_m_ value, revealed a 4-fold decrease in PUVA-treated fibroblasts (Figure 4(d)).

The increase of mitochondrial mass was confirmed by staining with MitoTracker Red, a dye that can be specifically sequestered into mitochondria to form a fluorescent aldehyde-fixable conjugate [20]. PUVA-treated fibroblasts showed a significant increase in mitochondrial mass compared to mock-treated control fibroblasts (Figures 4(a)–4(c)). The increased mitochondrial mass after PUVA might be due to high energy and biogenesis needs as a replenish mechanism in compensation for the reduced mitochondrial function, but the evidently decreased *ΔΨ*_m_ value indicated that a high percentage has defective electron transport chain. Interestingly, PUVA-induced mitochondrial mass increase and cell enlargement could be efficiently suppressed by feeding cells with ribose-5-phosphate (Figures 4(e) and 4(f)), further strengthens the idea that nucleotide synthesis might be the key event responsible for the metabolism change post-PUVA treatment.

### 3.6. PUVA-Induced ROS Caused an Early Onset of Replicative Senescence through Accelerated Telomere Attrition

**Figure 5:**
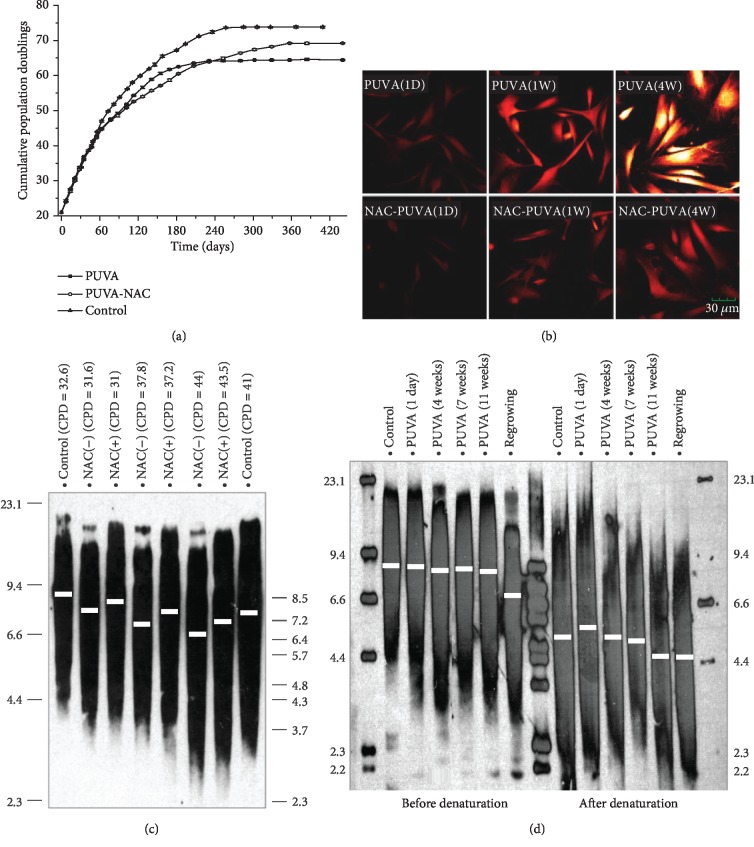
PUVA caused a decline of cell life span through oxidative telomere attrition. (a) Determination of proliferation capacity: starting from the same CPD, control fibroblasts (triangle), regrown fibroblasts treated with NAC (open circle) during the growth arrest stage, and regrown fibroblasts without NAC treatment (square) were passaged until reaching the replicative senescence stage. At each passage, cell numbers were determined in triplicates, and CPD were expressed as means ± SD. (b) Supplementation of NAC (5 mM) during the growth arrest stage post-PUVA treatment to suppress ROS. The medium was changed every three days to maintain sufficient amount of NAC. ROS were determined by DCF at day 2 after fresh supplementation of NAC. (c) Telomere length comparison between regrown and mock-treated fibroblasts at comparable CPD with or without NAC treatment during the growth arrest stage as determined by Southern blot using a 51-mer biotinylated telomeric probe and visualized by chemiluminescence. Mean TRF were calculated by the distributed fragment position, and the median position was marked with white line. (d) Determination of the average length of single-strand telomere by treating HinfI/RsaI digested genomic DNA with alkaline buffer to separate DNA double strands prior to electrophoresis and Southern blot.

The sustained ROS production following PUVA has an evident impact on the cell proliferation capacity. As measured by the cumulative population doublings (CPDs) [39], PUVA-treated fibroblasts reached the mitotic stationary stage of replicative senescence with a final CPD of 64.2. Compared to that of mock-treated fibroblasts with a total CPD of 73.8, 9.6 population doublings are decreased (Figure 5(a)). This substantially reduction of overall life span could be partly rescued by culturing PUVA-treated fibroblasts in medium supplemented with N-acetyl-L-cysteine (NAC), which is a GSH precursor and widely used as an antioxidant [40]. NAC was applied immediately after PUVA treatment, and the medium was refreshed every three days to ensure enough NAC supply until cells started to reproliferate. As shown in Figure 5(b), the ROS levels in PUVA-treated fibroblasts feeding with NAC were evidently decreased compared to that without NAC. The overall life span of PUVA-NAC fibroblasts is 69.2, increasing by 6 population doublings compared to that without NAC supplementation, though not fully restored to the level of control fibroblasts without PUVA treatment (Figure 5(a)).

To understand the underlying mechanism, ROS' impact on telomere was investigated.  Figure 5(c) shows the distribution pattern of the telomere length as determined by the terminal restriction fragments (TRF) using the Southern blot. Comparing the regrown fibroblasts post-PUVA treatment to that of fibroblasts without treatment at the comparable CPDs, the TRF of PUVA-treated fibroblasts (lanes 2, 4, and 6) were evidently shorter than that of mock-treated controls (Figure 5(c) lanes 1 and 8) as determined by the median position (marked with white line) as well as the top part which represent the longest telomere in the cell population with varied lengths of telomeres. The telomere lengths of PUVA-treated fibroblasts with NAC feeding (lanes 3, 5, and 7) were evidently protected compared to those without NAC, though they were still shorter than those of non-PUVA controls (lanes 1 and 8). These data indicate that PUVA-induced ROS is a major contributor of the accelerated telomere erosion and the reduced cellular life span.

#### 3.6.1. Accumulation of Single-Strand Breaks in Telomere during the Growth Arrest Stage

The telomere lengths were also determined during the growth arrest stage. As shown in Figure 5(d) (lanes 3 to 6), telomere lengths were constant at different time points, and there was no reduction comparing to mock-treated control fibroblasts (lane 2). However, a significant reduction of telomere length was observed in cells immediately after they started to reproliferate (lane 7). These data suggest that single-strand breaks (SSBs) may be accumulated in the telomere region during the growth arrest stage. To measure SSB, the same DNA samples (used in Figure 5(d) lanes 2 through 7) were denatured first before electrophoresis. As shown in Figure 5(d) lanes 9 to 14, the lengths of single-stranded telomeres showed a reduction with time during the growth arrest stage. The seemingly increased single-stranded TRF at day 1 post-PUVA treatment (lane 10) is apparently due to psoralen-DNA interstrand cross-links. By 11 weeks post-PUVA treatment, single-stranded telomere length was comparable to that of regrown fibroblasts. Thus, SSBs did accumulate in the telomere region and are responsible for the significant telomere length reduction in regrown fibroblasts.

## 4. Discussion

In the current study, we reported that a single PUVA treatment led to persistent endogenous production of ROS during the long-lasting growth arrest, which significantly reduced cellular life span by promoting accelerated telomere erosion. The elevated ROS level, which generated by malfunctioned mitochondria and activation of NADPH oxidase, was related to the repair of DNA bulky damages and through the activation of the pentose phosphate pathway. These results for the first time revealed an important link on how DNA damage impacted cell metabolism and subsequently contributed to premature aging.

The molecular mechanisms underlying the long-term side effects of PUVA therapy such as squamous cell carcinoma and premature skin aging have been generally assumed as the toxic effects of PUVA-induced DNA interstrand cross-links [11]. However, how the cellular level DNA damages affected organism/tissue aging has been poorly understood. Our data here give new insights on this question:

First, permanent changes were induced in the telomere region leading to an early onset of cellular senescence. A significant reduction in telomere length (~1-1.5 kb) in regrown fibroblasts post-PUVA treatment compared to mock-treated fibroblasts at the same CPD. This substantial difference was generated at the very beginning of regrowth, and our data support the idea that it was due to SSB accumulation in telomere—either inefficient repair or constantly generation. It would lead to partial telomere loss during DNA replication when newly synthesized strand meets the SSB to form a DSB resulting in replication fork collapse. In this study, ROS has been identified as a major factor responsible for the accelerated telomere attrition, since suppressing ROS by NAC could evidently prevent telomere erosion as well as partially restored the cellular life span. These data are in line with previous results using other oxidative damage models and confirmed the idea that oxidative stress is an important determinant for telomere length [41, 42].

Second, the persistent production of ROS would affect not only the cells where they were generated but also the intercellular microenvironment and adjacent cells in the same tissue due to the diffusion capability of certain ROS (e.g., H_2_O_2_), thus magnify the detrimental effects of DNA damage and spread to the whole tissue. In fact, we and others had shown previously that PUVA treatment led to an increased expression of matrix-degrading enzymes such as collagenase and stromelysin, which could lead to a loss of tissue integrity and elasticity commonly observed in skin aging [18, 43]. Buildup of chronic stress is known to be significant for the aging particularly in organism level, which is now termed senescence-associated secretory phenotype (SASP) [44–46]. The current study supports the idea that endogenous and persistent production of ROS might be a major contributor on the premature skin aging effects associated with PUVA treatment.

While the current study focuses mainly on the premature aging effects caused by PUVA, it should note that the elevated ROS level may also contribute to the increased incidence of squamous cell carcinoma by inducing mutations in critical genes, e.g., the tumor suppressor gene p53. A previous study detected increased occurrence of p53 mutation in specimens from PUVA-treated patients [47]. Interestingly, the expected psoralen-type mutations at AT sites were not observed, suggesting that mutation is likely not arisen from direct PUVA damage. The results of high level endogenous ROS production reported in the present study provided a new explanation to those results.

The present study suggests that elevated ROS level after PUVA was a response to the repair of bulky DNA damages, since ROS could also be induced by cisplatin and doxorubicin but less noticeable when treated with MMS. It is estimated that ~150-200 ICLs and 50-70 monoadducts per 10^6^ nucleotides were produced upon the PUVA dose used in the current study [48]. The persistent formation of H2AX and upregulation of ATM all pointed to the involvement of DSB repair being responsible for the production of ROS. Since BRCA1, a key player for HR, appeared to be negatively correlated with the elevation of ROS, whereas ROS was suppressed by the combined inhibition of DNA-PK and PARP1, indicating that ROS might be activated by NHEJ pathway. The above conclusion was further confirmed by the upregulation of 53BP1 and PP2A, which both inactivate HR and facilitate NHEJ through different mechanisms [32, 35]. ROS elevation might also be related to the repair of telomere damages where NHEJ plays a pivotal role, as our results demonstrated telomere being a major target of PUVA, and SSBs were accumulated during the growth arrest stage. This is predicated since the human telomere contains the TA base pairings that are prime targets of 8-methoxypsoralen [49], and the tandem TTAGGG repeats make the formation of ICL more likely to occur in telomere. Costaining of H2AX with telomere-specific fluorescence in situ hybridization demonstrated that DNA damage foci at telomeres persisted much longer than intrachromosomal DNA regions following PUVA treatment [50]. Further studies are required to address this important question as well as the signaling events involving ATM and PP2A, etc., and such information is crucial for elucidating the biological role and the underlying mechanisms of DNA damage-induced ROS stress response.

As to the downstream signaling events leading to ROS production, our data indicate that PUVA induced the activation of the PPP. This is in line with a previous study showing that upregulation of ATM in response to DNA damage could promote PPP flux [51]. The PPP is the major metabolic pathway to provide precursors for nucleotide biosynthesis, which generates ribose-5-phosphate (R5P) from glucose. Although the PPP has a major function of providing reducing molecules (NADPH and GSH) to defeat oxidative stress [52], results in the current study suggest that excess production of NADPH, on the contrary, may elevate ROS production through activating NOX since supplying high concentration of NADPH could generate ROS in control cells and suppress the endogenous ROS of PUVA-treated cells when adding NADP^+^. Blocking the whole PPP by inhibiting G6PDH, a rate-limiting enzyme involved in the oxidative branch of the PPP that convert NADP^+^ to NADPH, however, could not suppress ROS in PUVA-treated fibroblast. Together with the fact that mitochondrial mass was significantly increased after PUVA treatment, these data suggest there are high energy and biosynthesis needs in dealing with DNA repair. In this regard, NOX activation might play a role in maintaining a sufficient source of NADP^+^ to facilitate the ongoing of the PPP at the expense of generating excess ROS. The above idea is supported by the results that supplement PUVA-treated cells with the nucleotide precursor R5P could efficiently suppress ROS production and mitochondrial mass increase.

**Figure 6:**
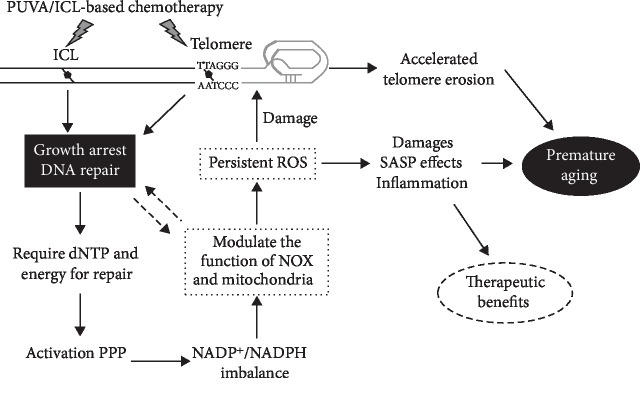
A proposed pathway for PUVA-induced ROS generation and its biological impacts. PUVA or similar chemotherapy drugs can induce DNA ICL especially in the telomere region. Owing to the high needs for energy and dNTP for DNA synthesis during DNA repair, the PPP was stimulated leading to imbalanced NADPH/NADP^+^ which then upregulated the expression and activity of NOX as well as mitochondrial biogenesis, causing persistent elevation of endogenous ROS level. The generated ROS caused telomere erosion and might affect adjacent cells and tissues through SASP mechanism, therefore contributing to the occurrence of premature aging.

Collectively, here, we reported a novel mechanism contributing to PUVA-induced premature aging, where a persistent production of ROS being the major culprit. A scheme model on how PUVA triggers ROS production and the subsequent signaling pathway impacting multiple biological functions is presented in Figure 6. Although the current study was pursued using PUVA as a model, the underlying mechanism may be generally true for the long-term adverse side effects associated with a large category of chemotherapeutic reagents, including many cancer drugs. In addition, the current study predicts that supplying additional NADP^+^ and R5P or similar metabolism modulators that can facilitate biosynthesis and energy needs in dealing with DNA repair might be able to prevent the premature aging or carcinogenetic side effects of many chemotherapeutic drugs.

## Data Availability

The data used to support the findings of this study are available from the corresponding author upon request.

## Supplementary Materials

Supplementary Materials Supplemental Table 1: primers for qPCR. Supplemental Figure 1: the expression of the mitochondrial transcription factor mtTFA is suppressed in *ρ*0 cells. Human dermal fibroblasts were incubated with ethidium bromide at a concentration of 0.1 *μ*g/ml as described in Materials and Methods. One week after treatment, mtTFA expression was determined in Rho0 fibroblasts and mock-treated control fibroblasts by Western blot. Supplemental Figure 2: ROS could be induced by cisplatin and doxorubicin, but less evident when treated with MMS. Human dermal fibroblasts could also be growth arrested with a one-time treatment of cisplatin (40 *μ*M) or doxorubicin (0.5 *μ*M) for a few days, then cell death occurred. Increased level of ROS could be observed as determined by the fluorescent dye 2′-7′ dichlorodihydrofluorescein diacetate (DCF). Fibroblasts treated with MMS (0.05% for 10 min) did not show an evident increase of ROS. Supplemental Figure 3: the upregulation of 53BP1 in PUVA-treated fibroblasts. As a key player controlling the repair choice between NHEJ and HR, the upregulation of 53BP1 was consistent with the downregulation of BRCA1 with time after PUVA treatment, suggesting a process going on to suppress homologous recombination. Supplemental Figure 4: ROS level can be stimulated by high concentration of NADPH. (A) Human dermal fibroblasts were preincubated with medium containing 5 mM NADPH for 2 hours, then ROS production was assessed by DCF. (B) NADPH-induced ROS could be suppressed by the NOX inhibitor. AEBSF (100 *μ*M) were added into the medium containing NADPH, preincubated for 2 hours before ROS determination. Supplemental Figure 5: mitochondrial membrane potential (*ΔΨ*_m_) determination by FACS analysis. Control, PUVA-treated cells at different time points (4 days, 16 days, and 11 weeks) and regrown cells were stained with 5 mg/ml of a polarization-sensitive dye JC-9 (MoBiTec, Goettingen) in PBS and then determined for red and green fluorescence by FACS (more details were described in Materials and Methods).
