# Supplementary material for: Slowly Repaired Bulky DNA Damages Modulate Cellular Redox Environment Leading to Premature Senescence
Source: Oxid Med Cell Longev. 2020 Feb 10;2020:5367102. doi: 10.1155/2020/5367102 (PMC7035574; doi:10.1155/2020/5367102)
Supplement: Supplementary Materials — Supplemental Table 1: primers for qPCR. Supplemental Figure 1: the expression of the mitochondrial transcription factor mtTFA is suppressed in ρ0 cells. Human dermal fibroblasts were incubated with ethidium bromide at a concentration of 0.1 μg/ml as described in Materials and Methods. One week after treatment, mtTFA expression was determined in Rho0 fibroblasts and mock-treated control fibroblasts by Western blot. Supplemental Figure 2: ROS could be induced by cisplatin and doxorubicin, but less evident when treated with MMS. Human dermal fibroblasts could also be growth arrested with a one-time treatment of cisplatin (40 μM) or doxorubicin (0.5 μM) for a few days, then cell death occurred. Increased level of ROS could be observed as determined by the fluorescent dye 2′-7′ dichlorodihydrofluorescein diacetate (DCF). Fibroblasts treated with MMS (0.05% for 10 min) did not show an evident increase of ROS. Supplemental Figure 3: the upregulation of 53BP1 in PUVA-treated fibroblasts. As a key player controlling the repair choice between NHEJ and HR, the upregulation of 53BP1 was consistent with the downregulation of BRCA1 with time after PUVA treatment, suggesting a process going on to suppress homologous recombination. Supplemental Figure 4: ROS level can be stimulated by high concentration of NADPH. (A) Human dermal fibroblasts were preincubated with medium containing 5 mM NADPH for 2 hours, then ROS production was assessed by DCF. (B) NADPH-induced ROS could be suppressed by the NOX inhibitor. AEBSF (100 μM) were added into the medium containing NADPH, preincubated for 2 hours before ROS determination. Supplemental Figure 5: mitochondrial membrane potential (ΔΨm) determination by FACS analysis. Control, PUVA-treated cells at different time points (4 days, 16 days, and 11 weeks) and regrown cells were stained with 5 mg/ml of a polarization-sensitive dye JC-9 (MoBiTec, Goettingen) in PBS and then determined for red and green fluorescence by FACS (more details were desc [file 5367102.f1.pdf]

Supplemental Info

Supplemental Table 1: Primers for qPCR

Gene	Forward	Reverse
18s	CACGGGAAACCTCACCCGGC	CGGGTGGCTGAACGCCACTT
PP2A	TGTCCGAGTCCCAGGTCAAG	TGCCACCAATTCTAAACAGTTCC
BRCA1	TGAAGACTGCTCAGGGCTAT	GTTAGAAGGCTGGCTCCC
NOX4	CAGGAGAACCAGGAGATTG	CTGAGAAGTTGAGGGCATT
ATM	TTACGGGTGTTGAAGGT	TCAGAGTAGGGTGAAGCT
mtDNA	CCTAGGGATAACAGCGCAAT	TAGAAGAGCGATGGTGAGAG

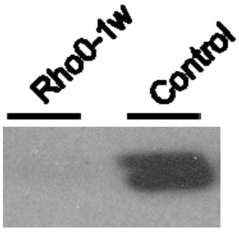

Supplemental Figure 1. The expression of the mitochondrial transcription factor mtTFA is suppressed in ρ0 cells. Human dermal fibroblasts were incubated with ethidium bromide at a concentration of 0.1 µg/ml as described in Materials and Methods. One week after treatment, mtTFA expression were determined in Rho0 fibroblasts and mock-treated control fibroblasts by Western blot.

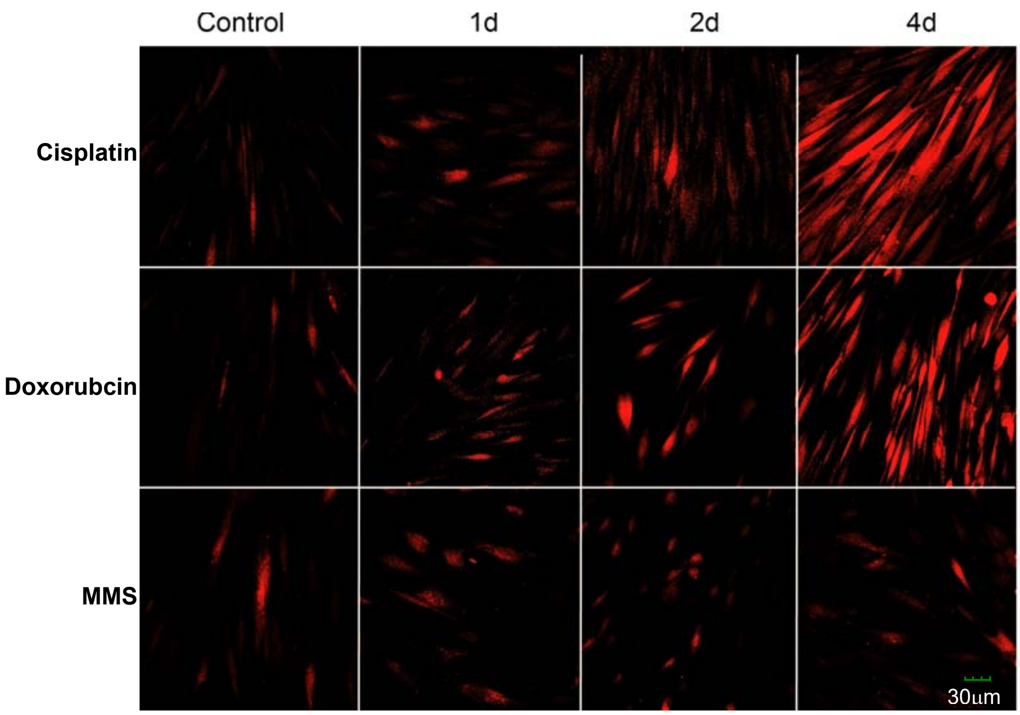

Supplemental Figure 2. ROS could be induced by cisplatin and doxorubicin, but less evident when treated with MMS. Human dermal fibroblasts could be also be growth arrested with a one time treatment of cisplatin (40μM) or doxorubicin (0.5μM) for a few days, then cell death occurred. Increased level of ROS could be observed as determined by the fluorescent dye 2’-7’ dichlorodihydrofluorescin diacetate (DCF). Fibroblasts treated with MMS (0.05% for 10min) did not show evident increase of ROS.

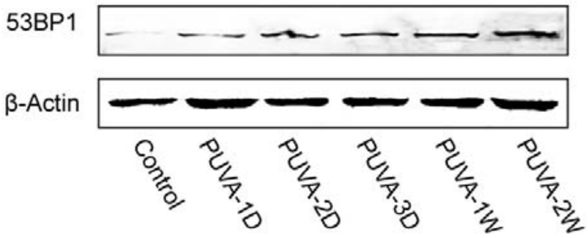

Supplemental Figure 3. The up-regulation of 53BP1 in PUVA-treated fibroblasts. As a key player controlling the repair choice between NHEJ and HR, the up-regulation of 53BP1 was consistent with the down-regulation of BRCA1 with time after PUVA treatment, suggesting a process going on to suppress homologues recombination.

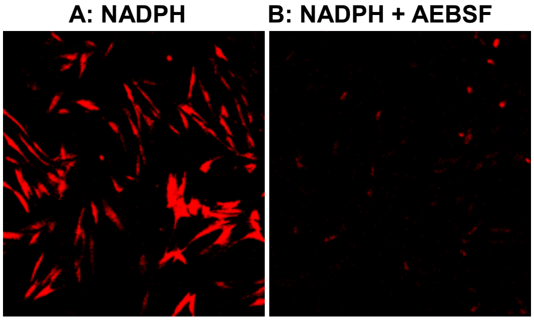

Supplemental Figure 4. ROS level can be stimulated by high concentration of NADPH. A) Human dermal fibroblasts were pre-incubated with medium containing 5mM NADPH for 2 hours, then ROS production were assessed by DCF. B) NADPH-induced ROS could be suppressed by the NOX inhibitor. AEBSF (100μM) were added into the medium containing NADPH, pre-incubated for 2 hours before ROS determination.

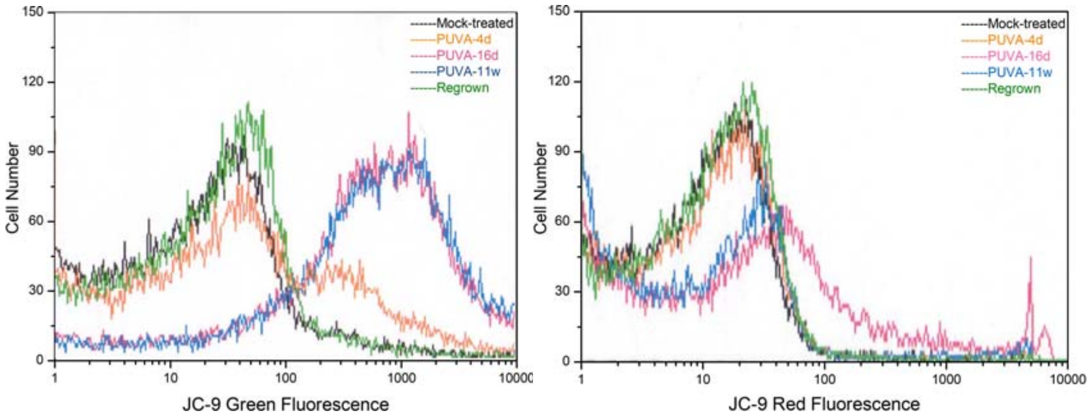

Supplemental Figure 5. Mitochondria membrane potential ($\Delta\Psi_m$) determination by FACS analysis. Control, PUVA-treated cells at different time points (4 day, 16 day and 11weeks) and regrown cells were stain with 5 mg/ml of a polarization-sensitive dye JC-9 (MoBiTec, Goettingen) in PBS, and then determined for red and green fluorescence by FACS (more details were described in Materials and Methods.
